# Critical ICP thresholds in relation to outcome: Is 22 mmHg really the answer?

**DOI:** 10.1007/s00701-024-05929-y

**Published:** 2024-02-05

**Authors:** Agnes C. Riparbelli, Tenna Capion, Kirsten Møller, Tiit I. Mathiesen, Markus H. Olsen, Axel Forsse

**Affiliations:** 1 Department of Neurosurgery, Copenhagen University Hospital – Rigshospitalet, Copenhagen, Denmark; 2 Department of Neuroanesthesiology, Copenhagen University Hospital – Rigshospitalet, Copenhagen, Denmark; 3 Department of Clinical Medicine, Faculty of Health and Medical Sciences SUND, University of Copenhagen, Copenhagen, Denmark; 4 Department of Clinical Neuroscience, Karolinska Institutet, Stockholm, Sweden

**Keywords:** ICP, TBI, Traumatic brain injury, Intracranial pressure, Threshold, Guidelines

## Abstract

**Purpose:**

Intensive care for patients with traumatic brain injury (TBI) aims, among other tasks, at avoiding high intracranial pressure (ICP), which is perceived to worsen motor and cognitive deficits and increase mortality. International recommendations for threshold values for ICP were increased from 20 to 22 mmHg in 2016 following the findings in a study by Sorrentino et al., which were based on an observational study of patients with TBI of averaged ICP values. We aimed to reproduce their approach and validate the findings in a separate cohort.

**Methods:**

Three hundred thirty-one patients with TBI were included and categorised according to survival/death and favourable/unfavourable outcome at 6 months (based on Glasgow Outcome Score—Extended of 6–8 and 1—5, respectively). Repeated chi-square tests of survival and death (or favourable and unfavourable outcome) vs. high and low ICP were conducted with discrimination between high and low ICP sets at increasing values (integers) between 10 and 35 mmHg, using the average ICP for the entire monitoring period. The ICP limit returning the highest chi-square score was assumed to be the threshold with best discriminative ability. This approach was repeated after stratification by sex, age, and initial Glasgow Coma Score (GCS).

**Results:**

An ICP limit of 18 mmHg was found for both mortality and unfavourable outcome for the entire cohort. The female and the low GCS subgroups both had threshold values of 18 mmHg; for all other subgroups, the threshold varied between 16 and 30 mmHg. According to a multiple logistic regression analysis, age, initial GCS, and average ICP are independently associated with mortality and outcome.

**Conclusions:**

Using identical methods and closely comparable cohorts, the critical thresholds for ICP found in the study by Sorrentino et al. could not be reproduced.

**Supplementary Information:**

The online version contains supplementary material available at 10.1007/s00701-024-05929-y.

## Introduction

Monitoring the intracranial pressure (ICP) of unconscious patients after traumatic brain injury (TBI) to detect secondary injury and evaluate treatment is standard practice in neurointensive care [2, 12, 30, 40]. An elevated ICP, if untreated, is thought to worsen motor and cognitive deficits and may be associated with increased mortality [2, 14, 40]. Consequently, intensive care management among other things aims at avoiding exposure to high ICP [12, 22, 47]. Normal ICP values are usually below 16 mmHg [3, 29, 31], although higher values can occur without consequences in the brain of a fully awake and orientated patient [8]. Given the frequent use of ICP monitoring in neurointensive care and its known association with outcome, ICP has become an independent treatment parameter and therapeutic targets have developed organically, despite the absence of randomised trials [12, 21, 27].

Until 2016, the internationally recognised treatment strategy for patients admitted to a neurointensive care unit (neuro-ICU) was to aim for an ICP below 20 mmHg [6, 44]. However, in 2012 Sorrentino et al. reported that an ICP limit of 22 mmHg provided a more optimal distinction between survival vs. death as well as between favourable vs. unfavourable outcomes at 6 months after traumatic brain injury (TBI). This suggestion was based on a convenience cohort of 459 patients with traumatic brain injury undergoing continuous ICP measurement for at least 6 h. Repeated chi-square testing across different ICP thresholds (integers) was done against the resulting outcome distributions for ICP values above and below these limits. Finally, a limit of 22 mmHg was found to produce the highest chi-square value, which was interpreted as providing the best discrimination between outcomes. Subsequently, management recommendations published by the International Brain Trauma Foundation regarding intracranial pressure were modified and a new target of “ICP below 22 mmHg” was adopted in guidelines [6]. Thus, the third edition of the International Brain Trauma Foundation guidelines stated that “treatment should be initiated with ICP levels above 20 mmHg”[44] while the updated fourth edition stated that “treating ICP above 22 mmHg is recommended because values above this level are associated with increased mortality” [6].

The updated guidelines were criticised for several reasons [26, 30, 33]. First, the calculations by Sorrentino et al. used mean ICP values for the entire monitoring period rather than fluctuations or spikes, which may not be the most important ICP-derived variable for establishing a relationship to outcome [19, 39]. Second, a defined threshold might lead clinicians to focus solely on ICP measurements instead of the global clinical context [33]. Both arguments suggest that clinical management must consider a more complex reality than single ICP values, while reproducibility and traceability of the suggested threshold have never been addressed. The findings by Sorrentino et al. reflect a cohort of patients with severe TBI from their catchment area, who were managed with a 20 mmHg ICP threshold. It is increasingly recognised that single observational datasets may be impossible to reproduce [18]; hence, such findings should be externally validated in independent populations before they are used for guidelines [13, 32, 36]. Finally, the report from Sorrentino et al. was based on an observational study; the relationship between a higher ICP and a worse outcome could in theory be due to bias by indication, meaning that it was harder to reduce an increased ICP in patients with more serious injuries and a more serious diagnosis. Thus, a strong relationship between ICP higher than a given threshold and a poor outcome would not necessarily imply that reducing the ICP below the threshold would improve outcome.

In the present study, we replicated the approach by Sorrentino et al. [38] in order to validate their findings with regard to threshold ICP values and risk factors for mortality and functional outcome. For this purpose, we included a cohort of patients from Eastern Denmark who were admitted to the neuro-ICU with TBI. As the primary outcome, we studied ICP thresholds as uniform discriminators between favourable and unfavourable outcomes for the full population and across subgroups, as suggested by Sorrentino et al.

## Materials and methods

### Patients

**Fig. 1:**
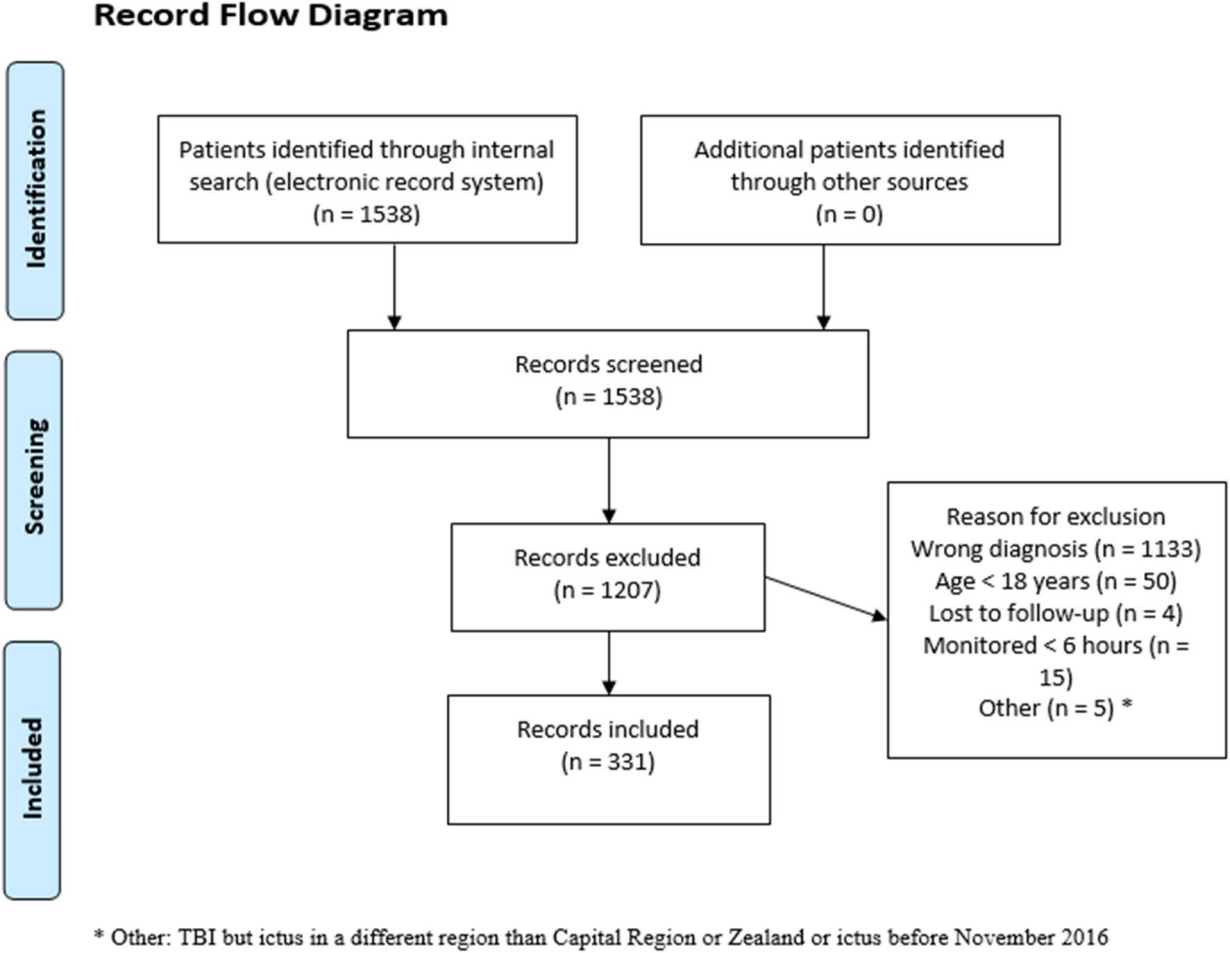
Record flow diagram

Because this retrospective study intended to validate the study by Sorrentino et al. [38], we applied the same methodology wherever this was feasible. A list comprising all patients with ICP monitoring and a minimum of 5 individual ICP data points admitted to the neuro-ICU of Copenhagen University Hospital—Rigshospitalet, Copenhagen, Denmark from November, 2016, to March, 2022, was extracted via the electronic patient record system (*Sundhedsplatformen*, EPIC Systems Corporation, WI, USA). This yielded a total of 1538 patients. All patients with a diagnosis of TBI were then identified. Patients under the age of 18 at time of admission to neuro-ICU, patients admitted to neuro-ICU solely for post-operative care, and patients with less than 24 individual ICP data points (equalling 6 h of monitoring) were excluded, leaving a total of 331 patients. See Fig. 1 for a flow diagram of the consort.

The electronic records of these 331 patients were reviewed and data registered for the following data points: main diagnosis, date and time of injury/ictus, first recorded Glasgow Coma Score (GCS) by a healthcare professional, survival status at 6 months (dead, alive, or unknown), 6-month assessment date, outcome on the Glasgow Coma Scale—Extended (GOSE) [25], and, if relevant, date and cause of death.

### Data collection

ICP was monitored invasively using either the intraparenchymal Codman® ICP Express™ monitor (Integra® Lifesciences, NJ, USA) or from 2019 the combined intraparenchymal Spiegelberg® ICP-Monitor (Spiegelberg®, Hamburg, Germany) with intraventricular drain. For external strain gauge measurements, the zero-reference point was set at the external auditory meatus (EAM), but most ICP measurements were achieved with electronic probes referenced to atmospheric pressure [9, 20, 28]. The intraparenchymal ICP-probe was placed either in the non-dominant frontal lobe or in the cavity after surgery. For patients perceived to benefit from drainage of cerebrospinal fluid (CSF), the intraventricular Spiegelberg drain with ICP monitoring was placed. The dataset contains a non-specified mix of patients monitored with the abovementioned methods. The monitoring data were automatically imported into the medical record system *Sundhedsplatformen* (Epic Systems Corporation, WI, USA) via a patient monitoring system (Intellivue Patient Monitor, Philips, Amsterdam, The Netherlands). The ICP data were extracted from an electronic data management system (*Sundhedsplatformen*, EPIC Systems Corporation, WI, USA) as average values over 15 min and subsequently averaged over the entire monitoring period, pursuant to the study by Sorrentino et al.

All approvals were obtained prior to study start from the Danish Patient Safety Authority and the Danish Data Protection Agency, file number R-21034304. According to Danish law, informed consent was not necessary.

### Patient management

Elevated ICP was treated according to the guidelines from the International Brain Trauma Foundation, with the notable exception that treatment was initiated at the pre-2016 level 20 mmHg. The treatment goal was an ICP < 20 mmHg and a cerebral perfusion pressure (CPP) > 60 mmHg with a target SpO_2_ of 94–98%, PaO_2_ of 10–12 kPa, and PaCO_2_ between 4.5 and 5.5 kPa. Patients received sedation with propofol or midazolam, analgesia with remifentanil, fentanyl, or morphine, and muscle relaxation with rocuronium as clinically indicated. ICP-lowering treatment comprised increased doses of sedatives, analgesics, and muscle relaxants; elevation of headrest; targeted blood glucose management aiming at 8–10 mM; short-term hyperventilation (< 2 min); and external ventricular drain (EVD) placement. In refractory cases, hypertonic fluid therapy, targeted temperature management aiming at normothermia were added, and decompressive craniectomy and intravenous infusion of thiopental were considered.

### Statistical analysis

All statistical analyses were carried out and figures created using R (Version 4.2.2, R Core Team, Vienna, Austria) . A *p* value of < 0.05 was considered statistically significant.

Patients were dichotomised as dead/alive and having a favourable/unfavourable outcome at 6 months. For the latter, GOSE 6–8 was regarded as favourable and GOSE 1–5 as unfavourable outcome.

Using 2 × 2 tables, Pearson’s chi-square values were repeatedly calculated around ICP integers from 10 up to 35. Thus, patients with an average ICP throughout the monitoring period at or above the integer in question were classified as having “high” ICPs and those with an average ICP below the integer as having “low” ICPs. The chi-square value was then calculated for this distribution against the clinical outcome distribution (i.e., dead/alive and unfavourable/favourable outcome, respectively). The ICP integer returning the highest chi-square value was termed the “threshold value,” which was considered to give the most significant discrimination between outcomes. A primary sensitivity analysis was performed using an identical approach to calculate ICP thresholds for subgroups according to age (up to 55 years vs. 55 and over), sex (female or male), and first recorded GCS (≤ 8 and below or ≥ 9). In addition, a secondary sensitivity analysis was performed after including all patients with a minimum monitoring period of 75 min instead of 6 h.

Finally, multiple logistic regression was performed using identical variables as risk factors for mortality and functional outcome (ICP, sex, age, GCS), while testing for linearity of the logit as well as multicollinearity.

## Results

Of the 331 included patients, 88 (27%) were females and 243 (73%) were males. Median age was 57 [IQR: 37–69] and the median duration of ICP monitoring was 121.75 h [IQR 36.875–273, which corresponds to a median number of individual data points of 487 [IQR: 147.5–1092]. Six months after admission, 94 (28%) had died (GOSE 1), 216 (65%) had an unfavourable outcome (GOSE 1–5), and 115 (35%) had a favourable outcome (GOSE 6–8).

**Fig. 2:**
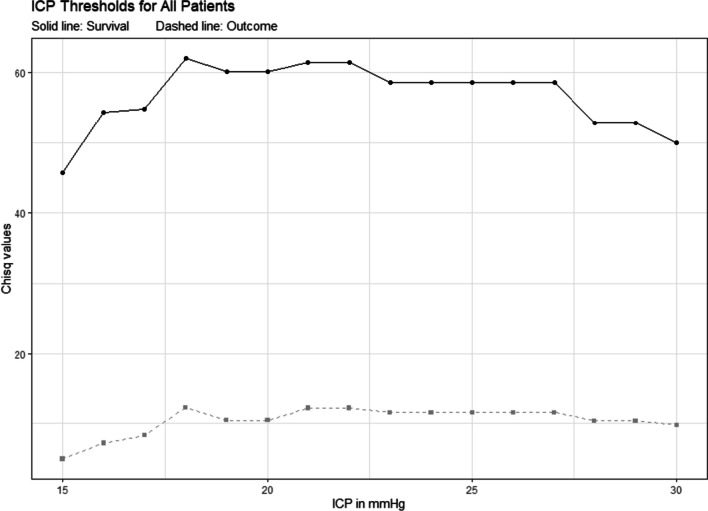
Chi-square values for mortality and outcome for the entire cohort. Chisq, chi-square; ICP, intracranial pressure

**Fig. 3:**
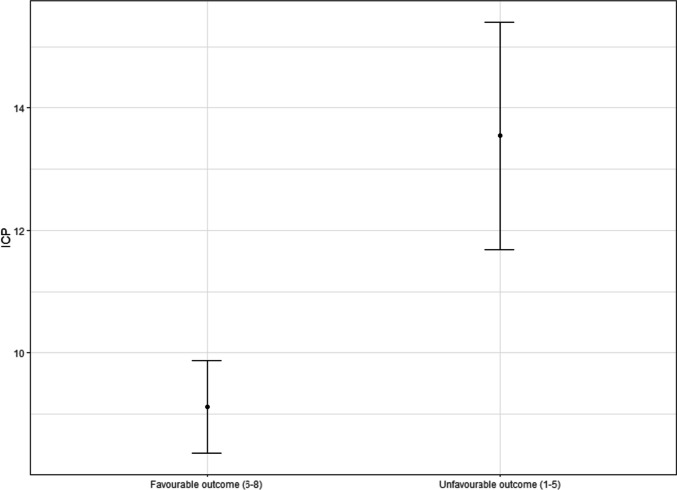
Mean ICP with 95% confidence intervals for patients with favourable (GOSE 6–8) and unfavourable (GOSE 1–5) outcome, respectively. ICP, intracranial pressure

The results for the calculated thresholds with their respective chi-square value, *p*-value, sensitivity, and specificity are seen in Table 1. Figure 2 depicts all calculated chi-square values for ICP values vs. mortality and outcome, respectively, for the entire patient cohort. An ICP of 18 mmHg yielded the highest chi-square value for mortality and outcome for the entire cohort. Figure 3 shows the mean ICP with 95% confidence intervals for favourable and unfavourable outcomes (including death) at 6 months.

**Table 1:** Thresholds for ICP in mmHg

	*N*	Threshold for survival in mmHg (X^2^, P)	Sensitivity (%) Specificity (%)	Threshold for favourable outcome in mmHg (X^2^, P)	Sensitivity (%) Specificity (%)
All patients	331	18	29	18	13
		(62.00, *P* < 0.001)	99	(12.26, *P* < 0.001)	99
> 55 years	175	16	22	*NS*	
		(20.76, *P* < 0.001)	99		
< 55 years	156	19–30	45	18	16
		(56.61, *P* < 0.001)	100	(9.19, *P* = 0.002)	100
Females	88	18–20	14	*NS*	
		(5.64, *P* = 0.018)	100		
Males	243	21–22	31	21–22	13
		(53.8, *P* < 0.001)	100	(10.29, *P* = 0.001)	100
GCS < 8	211	18	34	18	16
		(43.59,* P* < 0.001)	99	(7.95, *P* = 0.001)	100
GCS > 9	120	21–30	10	*NS*	
		(4.93, *P* = 0.027)	100		

X^2^ chi-square value, *P P*-value, *GCS* Glasgow Coma Scale, *NS* not significant

Thresholds for ICP values in mmHg. If a range is listed, e.g., 18–20 mmHg for female patients, the numbers 18, 19, and 20 all yielded equal chi-square values

The ICP thresholds for survival and functional outcome in subgroups varied between 16 and 22 for the older, female, male, and low GCS, and between 19 and 30 for the young and high GCS (Table 1). No threshold achieved statistical significance for functional outcome in the subgroups for older, female, and high GCS. Generally, chi-square values were three to six times as high for survival compared to functional outcome. Furthermore, the sensitivity and specificity calculations showed a low sensitivity for all the results (10–45%), while the specificity was very high (99–100%).

The secondary sensitivity analysis with a monitoring period of minimum 75 min included 15 extra patients of which seven were dead at 6 months, three were alive with an unfavourable outcome, and six had a favourable outcome. It yielded a global threshold of 27 mmHg and show the ICP thresholds for survival and functional outcome in subgroups varied between 27 and 30 mmHg for the young, male, and high GCS, and between 15 and 18 mmHg for the older, female, and low GCS (Supplemental materials).

Tables 2 and 3 show the results of the multivariable logistic regression analysis. Age, GCS, and mean ICP were significant predictors for both mortality and outcome, while sex was not significantly related to either mortality or outcome.

**Table 2:** Multiple logistic regression analysis for prediction of survival

	*B*	SE	*Z*	*P* value
Age	0.051	0.0095	5.388	< 0.001
Sex	0.164	0.337	0.486	0.627
GCS	− 0.152	0.038	− 3.963	< 0.001
Mean ICP	0.132	0.028	4.684	< 0.001

*B* regression coefficient, *SE* standard error, *Z* *Z* score, *GCS* Glasgow Coma Scale, *ICP* intracranial pressure

**Table 3:** Results of multiple logistic regression analysis for prediction of favourable outcome

	*B*	SE	*Z*	*P* value
Age	0.031	0.0073	4.23	< 0.001
Sex	0.281	0.286	0.982	0.3262
GCS	− 0.2	0.032	− 6.144	< 0.001
ICP	0.066	0.026	2.515	0.0119

*B* regression coefficient, *SE* standard error, *Z* *Z* value, *GCS* Glasgow Coma Scale, *ICP* intracranial pressure

## Discussion

In 2012, Sorrentino et al. reported that an ICP value of 22 mmHg represented a threshold regarding survival and functional outcome in a convenience cohort of 459 patients with severe TBI [38]. Although Sorrentino et al. interpreted their study to support the current guidelines at the time of publishing, and although the value was derived from an observational single-centre study, the report was cited as part of the basis for a subsequent level II B recommendation from the International Brain Trauma Foundation to treat ICP values above 22 mmHg [6]. The present study aimed to replicate this study but obtained a threshold of 18 mmHg. Our subgroup analyses suggested lower thresholds in the patients who were older, female, or had a low admission GCS, and higher values for young patients and males. In contrast to the original study, we found limited to no indication of a biological plausibility of an “optimal discriminator,” as there was virtually no progression in chi-square values around the “maximum value.” The incidental peaks of 18, 21, and 22 mmHg also appeared to be somewhat arbitrary (Fig. 2).

The findings regarding sensitivity and specificity are not in themselves problematic. Our analysis implied a low sensitivity for survival (29%), whereas specificity for survival was high (99%). Given the high specificity (meaning that few patients survived with a mean ICP above 18 mmHg), and in the absence of high-quality randomised trials, it would be intuitively attractive to set the threshold at this value. However, we strongly oppose the notion that the present data could be used to dictate a threshold above which ICP values should be viewed as invariably dangerous and should be treated at all costs. Firstly, this was an observational study, which were not based on randomised data. Thus, outcome differences could be driven by other factors than the differences in ICP. Secondly, even if the findings were assumed to represent a true causal relationship between ICP and outcome, other ICP metrics than the mean ICP would probably also be important for ensuring a good outcome. Thirdly, other aspects of supportive treatment such as targeted brain oxygen management and surgical procedures may change the relationship between ICP and outcome.

We performed a secondary sensitivity analysis with a monitoring period of minimum 75 min, which further emphasises how easily the threshold can be influenced. The new limit meant that 15 more people were added to the dataset. This changed the global threshold from 18 to 27 mmHg. Of the 15 patients who were eliminated by the 6-h minimum, seven (46%) were dead at the 6-month follow-up. They probably represented some of the most severe cases, who were potentially unresponsive to treatment or those who may have succumbed despite treatment.

In the validation approach, we elected to focus on ICP thresholds and not on CPP or pressure reactivity index (PRx) which were both analysed in the original study, because ICP remains the most frequently used invasive measure of intracranial homeostasis, with widely adopted targets and limits for treatment [2, 41, 48]. As in the original study, a retrospective cohort was included. However, our patient population was admitted from 2016 to 2022 (contrary to the original report, which studied patients admitted between 1992 and 2009); the lower age limit for inclusion was 18 years (contrary to the original age limit of 14 years). Furthermore, our approach differed with respect to the intervals for which ICP was averaged (15 min vs. 1 min in the original study). We used the extended 8-point version, GOSE, rather than the 5-point Glasgow Outcome Scale (GOS) for assessing functional outcome, as GOSE is recommended for research purposes for monitoring outcome after TBI [23, 25, 49]. It was developed from the GOS and translates to the original [50], which makes our dichotomised outcome comparable to the original study. Finally, the clinical management of our cohort differed from the original cohort with respect to the target range for PaCO_2_ (4.5–5.5 vs. 4–4.5 in the original study) and the target SpO_2_ (92% vs. 93%). As a result, our sample size was somewhat lower than the original sample size (331 vs. 459 patients); however, the number of patients older than 55 years was larger in our study (175 vs. 77), meaning that the lower sample size likely did not contribute to the fact that we could not identify statistically significant thresholds for the subgroups of older, females, and high GCS, or that the entire cohort retained the threshold of 18 mmHg that was identified in the female and low GCS. The overall heterogeneity of subgroup sizes mimics the subgroup sizes found in the Sorrentino study with fewer females than males and fewer patients with high compared to low initial GCS, except for a more equal distribution of age. Importantly, the cohort studied by Sorrentino et al. included patients before and after the 2007 recommendation by the Brain Trauma Foundation [5]. Thus, the authors describe that patients were managed slightly different over time, with a more CPP-centred algorithm before 2009 and a more ICP-centred approach after 2009. The implication of this is that the relationship between ICP and clinical outcome may also have shifted over time. By the same token, from 2018 some of the patients included in our study were managed according to both ICP and brain tissue oxygen tension.

The use of mean ICP to calculate the thresholds received criticism [26, 30] as severe TBI tends to be associated with long durations of ICU and hospital stay [45, 51] and, by inference, durations of ICP monitoring. As ICP can vary substantially over time, the subsequent monitoring period might encompass a period with ICP values in the normal area and as a result, the mean ICP alone might reflect a tail of normalised ICP values. An additional point of controversy is how “elevated ICP” and “mean ICP” should be understood. Sorrentino et al. justified their use of mean ICP in their limitations, and referenced a study from their own group, that showed an association between protracted elevated ICP and outcome [7]. That study defined a protracted episode as 30–40 min with an ICP above 40 mmHg, and the authors argued that ICP elevations of shorter duration would not affect mean ICP and thereby fail to affect outcomes in the 2012 study. We cannot judge from available data whether the argument reflects empirical data or an element of circularity. In contrast to Sorrentino et al., Bennis et al. analysed ICP spikes over 30 mmHg for only 3 min which they considered signs of disturbed autoregulation that worsened outcome [4]. Currently, empirical justification for either view appears insufficient. These examples show the need for more empirical data on the association between outcome and an increased ICP over time, and it would seem reasonable to suggest looking into threshold values based on fluctuations of ICP, time of exposure to high ICP, and treatment interventions for ICP [16, 23].

The fact that it was not possible to reproduce the threshold found by Sorrentino et al. questions whether a definitive threshold or a “one-size-fits-all” approach to ICP management is feasible [23, 26, 41]. Besides the obvious problems of basing a threshold on an observational single-centre study, we suggest that the identification of an “optimal ICP threshold” [17] for clinical purposes by identifying the point of greatest discrimination between outcomes is fundamentally flawed. Single-target values or ranges for ICP may not reflect what is physiologically desirable at definite times after injury [6] and the value of “optimal” is not easily translated to patient benefit or absolute chance of survival [4, 11, 42]. The idea of a fixed ICP threshold, above which all patients should at all times and in the context of all possible treatments, undergo ICP-reducing therapy during their admission, ignores the heterogeneity of clinical presentations and mechanisms of TBIs, and coping mechanisms of the patients [10, 23, 41], and it is at best overly simple and at worst dangerous to patients. The risk of unintended severe ICP elevations with small changes in intracranial volume depends on the location on the intracranial volume-pressure curve. An ICP of 22 mmHg may in some patients be located on the steep part of the curve, where even small fluctuations in intracranial volume may lead to deleterious increases in ICP; this may render the clinical management of such a threshold unfeasible, if not impossible. It is also not obvious that a treatment target should aim at the steepest discriminating ICP rather than to a lower ICP, which may be even more beneficial after TBI. The threshold ICP at any given time should be considered in the context of the specific ICP-reducing therapy and its risk profile. Thus, ICP-reducing therapies that are associated with little or no risk of harm, such as elevating the head of the bed or keeping the head in a neutral position, are often administered to “all” patients, even those in whom ICP is low [1, 43, 46]. In contrast, if all non-invasive ICP-reducing therapies have been applied, and only decompressive craniectomy is left, the risk of harm associated with craniectomy means that the risk–benefit balance shifts toward a higher ICP [37]. Accordingly, the optimal ICP threshold depends on what treatment has been administered and which treatment is presently considered for that individual patient.

### Limitations

As the method of analysis used by as Sorrentino et al. was applied in this study, the limitations of their study also apply to our study. Besides the limitation of the use of mean ICP, these include 1) the variation of appropriate thresholds for intervention over the course of time or in different clinical settings, as the shared knowledge of clinicians involved in the care of TBI patients evolves; 2) the lack of a time component in the threshold; 3) the fact that these analyses were carried out on patients receiving treatment for elevated ICP; and 4) the dichotomisation of GOS/GOSE to group patients after favourable and unfavourable outcomes at 6 months. This was done to create the 2 × 2 tables needed for the chi-square analysis. Dichotomisation of the outcome scales is not recommended as it limits the statistical power of the scales and limits the applicability of outcome analysis on TBI patients [24, 35].

In both cohorts an intraparenchymal ICP monitor was used, which has a known risk of underestimating the ICP with several mmHg with respect to the gold-standard true centre-ICP measured intraventricularly according to the head position of the patient and anatomical landmark used as the zero-reference point [34, 52]. The use of different brands and models of invasive ICP monitors may also play a role in the discrepancies in results [9, 15] as well as a potential difference in measuring techniques. The original study by Sorrentino et al. [38] used the Codman Microsensor ICP transducer (Codman & Shurtlef, Inc., Randolph, MA), but does not mention where in the brain parenchyma the transducer is placed. Similarly, the present study used the Codman® ICP ExpressTM monitor (Integra® Lifesciences, NJ, USA) and the Spiegelberg® ICP-Monitor (Spiegelberg®, Hamburg, Germany) and the specific placement of ICP monitor was not considered in this study. However, for the purpose of replicating the original study, we believe that our methods are comparable as both studies run the risk of underestimating the true centre ICP.

## Conclusion

We could not reproduce the ICP threshold of 22 mmHg proposed by Sorrentino et al. Using a similar approach, we found ICP threshold values of 18 mmHg for survival and outcome for the entire cohort, as well as the female subgroup and the subgroup of patients with a low initial GCS ≤ 8. For the remaining subgroups, the ICP threshold for both mortality and favourable outcome was 16–30 mmHg. Age, initial GCS, and mean ICP, but not sex, were independent risk factors for functional outcome and survival.

The development of ICP thresholds as treatment indicators for mortality and outcome and creation of guidelines are interesting research fields, but as illustrated in this study contain many pitfalls, which require further examination with various analytical methods. In the light of our results, it does not seem reasonable to recommend ICP thresholds based on the present application of statistical analyses, and it would seem imprudent not to question the relevance of the change of an ICP threshold from 20 to 22 mmHg in the 2016 International Brain Trauma Foundation guidelines and the consequences it may have on the treatment and research of TBI patients. Moreover, our analyses reflect an underlying heterogeneity of the populations that casts a shadow of doubt on the project of defining treatment thresholds that should apply across large populations of intensive care patients.

## Supplementary Information

Below is the link to the electronic supplementary material.

Supplementary file1 (DOCX 44 KB)

## Data Availability

The data and material that supports this study can be shared by the corresponding author upon request, with the notable exception that it will be anonymised, as it contains sensitive personal information of the participating patients.

## Abbreviations

CPP: Cerebral perfusion pressure
CSF: Cerebrospinal fluid
EAM: External auditory meatus
GCS: Glasgow coma score
GOS: Glasgow Outcome Scale
GOSE: Glasgow Coma Scale—Extended
ICP: Intracranial pressure
Neuro-ICU: Neurointensive care unit
PRx: Pressure reactivity index
TBI: Traumatic brain injury
