# Supplementary material for: Critical ICP thresholds in relation to outcome: Is 22 mmHg really the answer?
Source: Acta Neurochir (Wien). 2024 Feb 5;166(1):63. doi: 10.1007/s00701-024-05929-y (PMC10844356; doi:10.1007/s00701-024-05929-y)
Supplement: Supplementary file 1 — Supplementary file1 (DOCX 44 KB) [file 701_2024_5929_MOESM1_ESM.docx]

**Supplemental materials**

**Table 4** Thresholds for ICP in mmHg with a 75-minute minimum monitoring period

|  | **N** | **Threshold for survival in mmHg (^2^, P)** | **Sensitivity (%)**  **Specificity (%)** | **Threshold for favourable outcome in mmHg (^2^, P)** | **Sensitivity (%)**  **Specificity (%)** |
| --- | --- | --- | --- | --- | --- |
| **All patients** | 346 | 27  (70.22, *P* < 0.001) | 28  100 | 27  (14.75, *P* < 0.001) | 16  98 |
| **> 55 years** | 184 | 16  (25.29, *P* < 0.001) | 27  98 | 16  (5.58, P = 0.02) | 15  98 |
| **< 55 years** | 162 | 27-30  (66.28, *P* < 0.001) | 50  100 | 27-30  (10.93, *P* < 0.001) | 18  100 |
| **Females** | 95 | 15-16  (17.59, *P* < 0.001) | 33  99 | *NS* | *NS* |
| **Males** | 251 | 27  (56.89, *P* < 0.001) | 31  100 | 27  (11.35, *P* < 0.001) | 17  98 |
| **GCS < 8** | 218 | 15-16  (52.38, 4 *P* < 0.001) | 39  99 | 18  (9.84, *P* = 0.001) | 18  100 |
| **GCS > 9** | 128 | 27-30  (9.93, *P* = 0.001) | 10  100 | *NS* | *NS* |

^2^: Chi-square value. P: P-value

Thresholds for ICP values in mmHg. If a range is listed, e.g., 27-30 mmHg for young patients, the numbers 27, 28, 29 and 30 all yielded equal chi-square values.

**Table 5** Multiple logistic regression analysis for prediction of survival with a 75-minute minimum monitoring period

|  | **B** | **SE** | **Z** | **P value** |
| --- | --- | --- | --- | --- |
| **Age** | 0.05 | 0.01 | 5.47 | < 0.001 |
| **Sex** | 0.1 | 0.33 | 0.3 | 0.34 |
| **GCS** | -0.17 | 0.04 | -4.27 | < 0.001 |
| **Mean ICP** | 0.13 | 0.03 | 5.01 | < 0.001 |

B: regression coefficient, SE: standard error, Z: Z score. GCS: Glasgow Coma Scale. ICP: Intracranial pressure

**Table 6** Results of multiple logistic regression analysis for prediction of favourable outcome with a 75-minute minimum monitoring period

|  | **B** | **SE** | **Z** | **P value** |
| --- | --- | --- | --- | --- |
| **Age** | 0.03 | 0.01 | 4.33 | < 0.001 |
| **Sex** | 0.2 | 0.28 | 0.72 | 0.34 |
| **GCS** | -0.2 | 0.03 | -6.33 | < 0.001 |
| **ICP** | 0.07 | 0.03 | 2.83 | 0.005 |

B regression coefficient, SE standard error, Z Z value, GCS: Glasgow Coma Scale. ICP: Intracranial pressure

**Figure 4** Chi-square values for mortality and outcome for the entire cohort with a 75-minute minimum monitoring period.


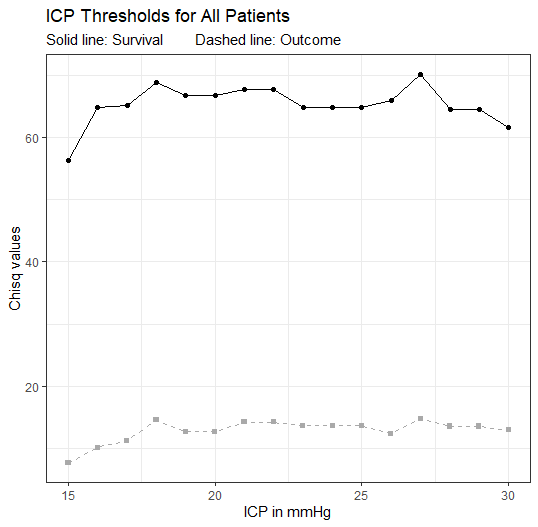


Chisq: Chi-square; ICP: intracranial pressure.

**Figure 5** Mean ICP with 95% confidence intervals for patients with favorable (GOSE 6-8) and unfavorable (GOSE 1-5) outcome, respectively, with a 75-minute minimum monitoring period


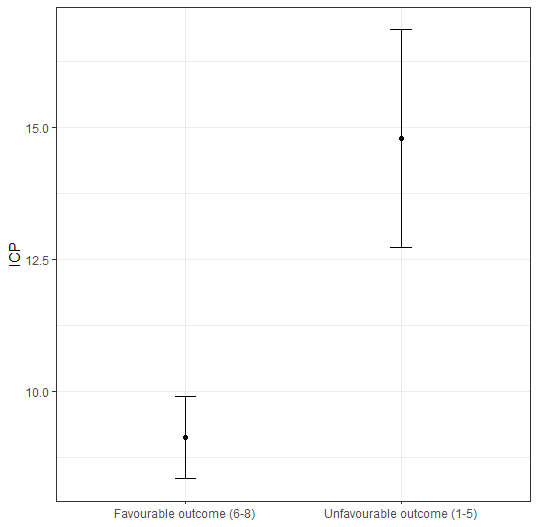


ICP: Intracranial pressure.
